# The Converging Pathologies of Obstructive Sleep Apnea and Atrial Arrhythmias

**DOI:** 10.7759/cureus.9388

**Published:** 2020-07-25

**Authors:** Sana Riaz, Harneet Bhatti, Parth J Sampat, Amit Dhamoon

**Affiliations:** 1 Internal Medicine, State University of New York Upstate Medical University, Syracuse, USA

**Keywords:** obstructive sleep apnea, continuous positive airway pressure, atrial arrhythmia, sudden cardiac death

## Abstract

Obstructive sleep apnea (OSA) is highly prevalent in the United States (US). Along with epidemic rates of obesity, the rate of OSA cases is also on the rise. OSA is associated with multiple chronic health conditions, including hypertension, diabetes, stroke, myocardial ischemia, and heart rhythm disturbances. OSA is commonly treated with continuous positive airway pressure (CPAP) therapy. Several reports indicate that effective treatment of OSA can reduce the risk of cardiovascular diseases, including cardiac arrhythmias, especially atrial fibrillation (AF). CPAP therapy helps to maintain sinus rhythm after interventions such as electrical cardioversion and catheter ablation in patients with AF. However, more data is required to establish a relationship between OSA and other atrial arrhythmias as well to evaluate the effect of CPAP. This review will compile the latest evidence on the pathophysiology, management, and treatment of atrial arrhythmias associated with OSA.

## Introduction and background

Obstructive sleep apnea (OSA) is a highly prevalent, yet underdiagnosed, disease in the United States (US) and across the world. OSA is characterized by multiple episodes of partial or complete upper airway collapse during sleep leading to a reduction or complete cessation of airflow. An estimated 15-20 million American adults suffer from OSA, a prevalence comparable to that of diabetes [1]. Approximately 20% of adults have at least mild OSA, and approximately 7% of adults have moderate-to-severe OSA. The prevalence of OSA is twice as high in men as in women, and the condition is more prevalent in obese individuals than normal-weight individuals [1].

There has been a constant rise in obesity rates in the US over the last several decades. The prevalence of obesity in the US has increased from 30.5% in 1999-2000 to 42.4% in 2017-2018. During the same time period, the prevalence of severe obesity increased from 4.7% to 9.2% [2]. A double-digit percent increase, ranging from 14-55%, has also been found in the prevalence of OSA when compared to data from 1988, suggesting a link between obesity and OSA [3]. OSA is associated with many metabolic and physiological consequences such as diabetes, dyslipidemia, and fatty liver disease, along with depression, accidents, headaches, and decreased quality of life [4,5]. Additionally, there has been an evolving recognition of the role of OSA in cardiovascular diseases [1]. OSA is associated with hypertension, heart failure, cerebrovascular accidents, myocardial infarction, atrial fibrillation (AF), and sudden cardiac death (SCD) [1].

The association between sleep apnea and arrhythmias is an emergent issue in cardiology [6]. Cardiac arrhythmias are relatively common and are associated with significant morbidity and mortality. AF is the most common cardiac arrhythmia. It affects an estimated 2.7-6.7 million people in the US. Its impact on healthcare is expected to rise dramatically in the coming decades as the number of cases is projected to rise from 5.6 to 15.9 million by 2050 [7]. The increasing number of people with AF, especially if untreated, can result in a further increase in deleterious health consequences, including heart failure and stroke. The majority of published literature to date focuses on the association between OSA and AF, and insufficient data is available on other atrial arrhythmias. This literature review will provide an overview of the latest evidence on the relationship between the pathophysiology, management, and treatment of OSA and atrial arrhythmias. We will also review the implications of continuous positive airway pressure (CPAP) therapy on patients with AF.

## Review

Obstructive sleep apnea and atrial fibrillation

AF is the most commonly sustained arrhythmia worldwide and affects nearly 33.5 million people globally [8]. AF is associated with more than 450,000 hospitalizations, and it contributes to more than 150,000 deaths annually [9]. The prevalence of OSA in patients with AF is 21-74% [10]. Several studies have confirmed the increased incidence of AF in patients with OSA. The Sleep Heart Health Study, which engaged in a comparison between 228 patients with severe OSA and 338 patients without OSA, found that 4.8% of patients with severe OSA had AF, while only 0.9% of patients without OSA had AF [11]. Additionally, a retrospective study by Gami et al. involving 3,542 patients showed that OSA is a strong predictor of the incidence of AF. The study also concluded that OSA is a strong predictor of AF with a hazard ratio (HR) of 2.18. It was noted that AF occurred in 4.3% of patients with OSA and 2.1% of patients without OSA. The study showed that the cumulative probability of AF was significantly higher for subjects who are <65 years of age with OSA compared with those without OSA [12]. Similar findings were noted in a more recent meta-analysis study involving a large study population of 19,837, which showed that the odds of having AF was two-fold higher in patients with OSA compared to those without OSA [13]. It has been noted that the risk of AF recurrence after catheter-based pulmonary vein isolation (PVI) is higher in patients with OSA [10,14]. These studies suggest that the prevalence of OSA is significantly higher in patients with AF compared to the general population [15].

Obstructive sleep apnea and other atrial arrhythmias

Several small observational studies have suggested a positive correlation between OSA and other arrhythmias such as atrioventricular (AV) block, sick sinus syndrome, and other supraventricular tachycardias. One of the largest and earliest studies by Guilleminault et al. assessed 400 patients with severe OSA using polysomnography and 24-hour Holter monitoring. They reported that bradyarrhythmias (i.e., second- or third-degree AV block or sinus pauses of >2 seconds) occurred in 18% of the study population. They also reported atrial tachycardia, paroxysmal AF, and paroxysmal atrial flutter in 7, 3, and 1% of the study population respectively. There was no control group in their study. They noted that these atrial arrhythmias were only present during sleep and not present when patients were awake, which demonstrates a likely link between OSA and the arrhythmias. In the same study, resolution of sleep time atrial arrhythmias was demonstrated in 50 patients after tracheostomy, suggesting that the arrhythmias were associated with sleep apnea [16]. More recently, an observational study (with no control group) by Becker et al. assessed 239 patients with OSA using polysomnography and 24-hour Holter monitoring and reported that 7.5% of these patients had significant bradyarrhythmias. Also, they found that the prevalence of bradyarrhythmias correlated strongly with OSA severity and degree of nocturnal desaturations [17].

The increased vagal tone noted with nocturnal hypoxemia increases susceptibility to bradycardic and conduction rhythm disorders in patients with OSA. This seems to occur more frequently during rapid eye movement (REM) sleep and with decreases in oxygen saturation of at least 4% [18]. Nocturnal hypoxemia, a hallmark of OSA, predicts SCD independently of well-established cardiovascular risk factors, linking SCD with OSA [17,19].

Even though many studies have demonstrated associations between atrial bradyarrhythmias, SCD, and OSA (Table 1), most of these studies were observational studies conducted with small sample sizes. Larger multicentric trials are required to confirm the correlation between OSA and atrial arrhythmias.

Pathophysiology of cardiac arrhythmia and obstructive sleep apnea syndrome

There are several hypotheses explaining the pathophysiology of cardiac arrhythmias related to OSA. OSA is characterized by a recurrent interruption in ventilation due to repetitive airway collapse, leading to apneic and hypopneic episodes. An apneic event is defined as a 90% reduction in flow with the presence of respiratory effort for 10 seconds or longer. To be characterized as a hypopneic episode, the following criteria must be met: i) the peak signal excursions drop by >30% of pre-event baseline using nasal pressure, positive airway pressure-derived flow, or an alternative hypopnea sensor, ii) the duration of the >30% drop in the signal excursion is >10 seconds, and iii) there is a >3% oxygen desaturation from pre-event baseline or the event is associated with an arousal. Intermittent apneic and hypopneic events lead to arterial desaturations and hypercapnia [20].

OSA is associated with both parasympathetic stimulation during early phases of apnea and sympathetic stimulation in the later phases of apnea; thus, both “vagotonic” and “adrenergic” arrhythmias can be triggered [21]. Hypoxia, through its effect on peripheral chemoreceptors in the carotid body, and hypercapnia, through its effect on the central chemoreceptors, cause increased activation of the sympathetic nervous system. On the other hand, increased intrathoracic negative pressure caused by forced inspiration in response to obstructed airways stimulates the vagus nerve, whereas hypoxemia in the setting of OSA induces the activity of the carotid body. Together, these stimuli can produce a transient increase in parasympathetic activity. The increased vagal tone with nocturnal hypoxemia increases susceptibility to bradycardic and conduction rhythm disorders. Arrhythmias occur more frequently during REM sleep, and with oxygen desaturations of 4% [18]. Nocturnal hypoxemia also predicts SCD independently of well-established cardiovascular risk factors [17,19], linking SCD with OSA.

In contrast to the idea that an increase in vagal tone in OSA leads to arrhythmias, another hypothesis links the increased sympathetic stimulation seen in OSA in later phases of apnea with these cardiac arrhythmias. Arousal following apneic episodes with re-establishment of respiration through cortical centers causes sympathetic discharge and a decrease in vagal tone, resulting in a marked increase in heart rate [22].

Another theory linking atrial arrhythmias to OSA is that increased vascular inflammation associated with OSA may predispose individuals to AF. OSA is associated with higher levels of interleukin-6 (IL-6) and tumor necrosis factor-alpha (TNF-α), which is thought to create a systemic proinflammatory state [23]. OSA is also known to cause oxidative stress and a state of hypercoagulability [23]. Studies have suggested that inflammation and oxidative stress can be associated with atrial remodeling, which can lead to AF [24].

An additional hypothesis focuses on the changes in intrathoracic pressure in individuals with OSA [23,25,26]. Breathing against a collapsed pharyngeal wall can lead to a substantial shift in the intrathoracic pressures which, when transmitted to the atrial chamber, can result in enlargement and can also lead to remodeling of the pulmonary vein ostia. Both of these changes can predispose one to arrhythmias, particularly AF [27].

Management

Continuous Positive Airway Pressure (CPAP)

The mainstay treatment of AF focuses on rate control, rhythm control, and anticoagulation for the prevention of complications. Despite these treatments, there remains a pressing need for alternative therapies to control AF. Given the association of OSA with AF, theoretically, the treatment of OSA may help with the management of AF. Studies have shown that CPAP may have favorable effects on patients with atrial arrhythmias and OSA by reducing the number of apneic events, thereby leading to increase in oxygen supply, reduction in sympathetic activity, and reduction in the exaggerated shift of intrathoracic pressure, which in turn reduces mechanical stretching and the subsequent electromechanical arrhythmogenic trigger [21]. CPAP therapy is also associated with a decrease in brain natriuretic peptide (BNP) levels, which is an indicator of the decreased cardiac wall stretching. These findings suggest reversibility in the arrhythmogenic substrates induced by OSA by treatment with CPAP [21]. Data from meta-analyses have shown that patients with OSA have a 25-31% increased risk for AF recurrence after catheter ablation in comparison with patients with no sleep apnea [28]. Another meta-analysis performed by Qureshi et al. [29] and Shukla et al. [28] in 2015 demonstrated reductions in recurrence of AF after CPAP use. In another meta-analysis by Li et al., the risk of recurrence of AF increased from 31% to 57% in patients with OSA not undergoing CPAP therapy [30]. It is also noteworthy that CPAP users were found to have the same risk of AF recurrence as patients without OSA [30]. Besides showing reductions in recurrence of AF in CPAP users compared to non-CPAP users, the study by Shukla et al. also demonstrated a similar rate of reductions in AF recurrence in CPAP users following PVI. These results indicated that the relative risk reduction in AF recurrence with CPAP users remained consistent irrespective of whether they were managed medically or with PVI, thereby suggesting the presence of alternate proarrhythmic mechanisms that may be controlled with CPAP use and may remain unchecked with the conventional treatment strategies for AF. These study findings are significant as they provide evidence to the clinician about an additional means to reduce AF recurrence in patients with OSA [29].

Other Treatment Modalities

CPAP is a highly effective treatment option for the management of OSA. However, issues with adherence can limit its efficacy. Compliant regular users are defined as those who meet Centers of Medicare and Medicaid Services (CMS) compliance guidelines (mean positive airway pressure use of ≥4 hours per night for more than 70% of nights). Common reasons for non-adherence are dermatitis, rhinitis, epistaxis, mask leak, aerophagia, barotrauma, and claustrophobia [31]. Based on the literature, the adherence range is 40-85%, which has persuaded healthcare professionals to explore alternative therapies for the management of OSA [31]. Upper airway stimulation, especially hypoglossal nerve stimulation, is a new and alternative therapy for patients with OSA who cannot tolerate CPAP therapy. Oral appliances such as tongue-retaining devices and mandibular advancement devices or surgical interventions have been used in patients who cannot tolerate or do not wish to utilize CPAP. Surgical options for OSA include tonsillectomy and nasal or palatal surgeries [32]. Weight-loss is another option since there is evidence of prevention and reduction of AF burden through aggressive risk-factor modifications [15].

Table 1 summarizes the studies showing the correlation between OSA and atrial arrhythmias. Based on the latest literature, CPAP has shown to be the most effective intervention to decrease the recurrence of atrial arrhythmias in patients with OSA (Table 2). However, more data is required to evaluate the therapeutic role of other options, as mentioned above, in the management of OSA and atrial arrhythmias.

**Table 1:** Studies showing the correlation between OSA and atrial arrhythmias OSA: obstructive sleep apnea; AF: atrial fibrillation; CI: confidence interval; HR: hazard ratio; CPAP: continuous positive airway pressure; BiPAP: bilevel positive airway pressure; AHI: apnea-hypopnea index; SAS: sleep apnea syndrome; SCD: sudden cardiac death; AV: atrioventricular; OR: odds ratio; LVEF: left ventricular ejection fraction

Name and year of study	Type of study	Number of participants	Purpose	Conclusions
Atrial fibrillation				
Feng et al., 2019 [33]	Retrospective study	506,604	To understand the effect of OSA on readmission rates and postoperative AF in patients following cardiac surgery	Administrative databases from five states were collected between 2007-2014; 32,545 had a diagnosis of OSA. It was found that 40.4% with OSA developed AF compared to 35.8% without a diagnosis of OSA (p<0.001). Participants with OSA were also noted to have high 30- and 90-day readmission rates
Youssef et al., 2018 [13]	Meta-analysis	19,837	To assess the relationship between OSA and the risk of AF	The analysis was conducted using nine studies. The control group included 7,582 subjects, and the OSA group included 12,255 subjects. The risk of AF was found to be higher among OSA versus the control group (OR: 2.120, 95% CI: 1.845–2.436, Z; 10.598; p<0.001)
Holmqvist et al., 2015 [34]	Observational	10,132	To determine if patients with OSA have a higher likelihood of progressing to more persistent forms of AF or required more hospitalizations and/or have worse outcomes compared to patients without OSA	It was noted that patients with OSA had a higher risk of hospitalization (43 vs. 35 events/100 patient-years, p=0.0078), but similar rates of mortality, AF progression, and major adverse cardiovascular events as patients without OSA
Ng et al., 2011 [35]	Meta-analysis	3,995	To investigate the role of OSA on AF recurrence after catheter-based pulmonary vein isolation	Patients with OSA have a 25% greater risk of AF recurrence after catheter ablation than those without OSA (risk ratio: 1.25, 95% CI: 1.08-1.45, p=0.003)
Gami et al., 2007 [12]	Retrospective cohort study	3,542	To identify obesity and OSA as independent predictors of incidence of atrial fibrillation/flutter	AF occurred in 114 of 2,626 patients (4.3%) with OSA and 19 of 916 patients (2.1%) without OSA. OSA (defined by an AHI of 5) was a strong predictor of incident AF (HR: 2.18, 95% CI: 1.34-3.54, p=0.002). It was also noted that the severity of OSA was associated with a higher incidence of AF. The study also indicated that the cumulative probability of incident AF was significantly higher in subjects of <65 years of age with OSA compared with those without OSA
Mehra et al., 2006 [11]	Longitudinal cohort	566	To determine the prevalence of nocturnal cardiac arrhythmias in sleep-disordered breathing patients	The study involved 228 patients with OSA and 338 patients without OSA. AF had a prevalence of 4.8% in the study population. It was determined that patients with severe OSA would have a two-to-four-fold higher chance of developing complex arrhythmias
Gami et al., 2004 [15]	Prospective, cross-sectional	524	To determine the prevalence of OSA in AF patients	This study involved 151 patients with AF and 312 patients with another cardiovascular disease. The prevalence of OSA was higher in patients with AF than in the opposing group. The adjusted OR was 2.19
Porthan et al., 2004 [36]	Case-control	115	To determine the prevalence of arrhythmias in OSA patients	OSA was common in AF patients. However, the study could not demonstrate that OSA was more common in AF patients than in the corresponding controls
Javaheri et al., 1998 [37]	Prospective	81	To determine the prevalence of arrhythmias in OSA patients	All patients were male, with stable heart failure and left ventricular ejection fraction (LVEF) of <45%. The prevalence of AF was 22% versus 5% (p=0.026) in this study population
Flemons et al., 1993 [38]	Prospective	263	To determine the prevalence of arrhythmias in OSA patients	Patients with sleep apnea were found to have a low prevalence of arrhythmias. There was a 1.3% prevalence of complex ventricular ectopy, a 2.6% prevalence of frequent ventricular premature beats, 1.3% prevalence of second-degree AV block, and a 5.2% prevalence of sinus arrest
Sick sinus syndrome				
Koehler et al., 1997 [18]	Prospective	16	To determine the correlation between sleep stage, oxygen desaturation, and apnea-associated bradyarrhythmias as well as to assess the effect of treatment with CPAP	During rapid eye movement (REM) sleep, the frequency of heart block was higher than during non-REM sleep (87.9% compared to 12.1; it was noted to be statistically significant); 93.5% of bradyarrhythmias occurred with desaturation of at least 4%. With CPAP/bilevel positive airway pressure (BiPAP) therapy, apnea/hypopnea index (AHI) decreased from 75.5 ±39.6·h-1 to 3.0 ±6.6·h-1 (p<0.01)
Simantirakis et al., 2004 [39]	Longitudinal	23	To determine the prevalence of arrhythmias in OSA patients	Twenty-three patients were studied using a loop recorder; 47% of these untreated patients had nocturnal bradyarrhythmias. On the contrary, bradyarrhythmias were noted in only 13% of the patients on a 48-hour Holter monitor
Garrigue et al., 2007 [40]	Observational	98	To determine the prevalence of sleep apnea syndrome (SSA) in pacemaker patients	The overall prevalence of sleep apnea syndrome (SAS) was 59% in pacemaker patients. In patients with sinus node dysfunction, 58% were noted to have clinically silent SAS, and 27% had severe SAS (AHI: >30/hour)
Steiner et al., 2008 [41]	Observational	12	To understand the relationship between sleep apnea and sinus abnormalities	The study included patients with symptomatic heart failure (New York Heart Association class II-III). There was no correlation noted between mild sleep apnea and sinus abnormalities
Atrioventricular block				
Becker et al., 1998 [17]	Observational	239	To determine the prevalence of heart block in patients with sleep apnea	It was reported that 7.5% of patients had significant bradyarrhythmias (i.e., second- or third-degree AV block or sinus pauses of >2 seconds). Also, it was noted that the prevalence of bradyarrhythmias correlated strongly with OSA severity and degree of nocturnal desaturation, such that all patients with significant bradyarrhythmias had an AHI 60/h or more (accounting for 20% of this subgroup of patients)
Sudden cardiac death				
Gami et al., 2013 [19]	Prospective, longitudinal	10,701	To determine if there is an association between SCD and OSA	SCD was best predicted by age of >60 years (HR: 5.53), AHI of >20 (HR: 1.60), mean nocturnal oxygen saturation of <93% (HR: 2.93) and lowest mean nocturnal oxygen saturation of <78% (HR: 2.60) (all p<0.0001)
Gami et al., 2005 [42]	Retrospective	112	To determine the rate of sudden death in OSA	People with sudden death from cardiac causes from midnight to 6 a.m. had a significantly higher AHI than those with sudden death from cardiac causes during other time intervals. The relative risk of sudden death from cardiac causes between midnight to 6 a.m. in patients with OSA was 2.57 (95% CI: 1.87-3.52)

**Table 2:** Studies showing the effect of CPAP on patients with atrial arrhythmias and OSA OSA: obstructive sleep apnea; AF: atrial fibrillation; CI: confidence interval; CPAP: continuous positive airway pressure; AV: atrioventricular

Name and year of study	Type of study	Number of participants	Conclusions
Becker et al., 1995 [43]	Observational	239	17 of the 239 patients with OSA were noted to develop heart block (7.1%). Following treatment with CPAP, AV block and sinus arrest were eliminated in 12 of the 17 patients. Episodes were reduced in another three patients. One patient continued to have increased frequency of sinus arrest after four weeks of CPAP treatment
Harbison et al., 2000 [44]	Prospective, cross-sectional	45	In this study, only eight patients (18%) demonstrated pathologically significant rhythm disturbances. Recurring sinus pauses during sleep were the most common dysrhythmia (six patients), lasting 10 seconds. One patient also developed an episode of a variable second-degree AV block lasting 4.5 minutes. The resolution of rhythm disturbances was noted in seven out of eight patients following treatment with CPAP. Additionally, the resolution of non-significant rhythm disturbances was also noted with CPAP
Kanagala et al., 2003 [45]	Observational	43	The recurrence of AF one year after electrical cardioversion was compared between patients with treated and untreated OSA. Maintenance of sinus rhythm was strongly associated with CPAP use, and only 42% of the treated patients had recurrent AF, compared with 82% of untreated patients
Simantirakis et al., 2004 [39]	Longitudinal	23	Eight weeks after initiation of treatment with CPAP, the total number of bradyarrhythmias tended to decrease. Six months after treatment, there was only one patient with bradycardias and one with pauses. No pauses were detected after the six-month time point and no bradycardias after the 10-month time point
Marin et al., 2005 [46]	Prospective, cohort	1651	Patients with the severe untreated disease had a higher incidence of fatal cardiovascular events (1·06 per 100 person-years) and non-fatal cardiovascular events (2·13 per 100 person-years) compared to untreated patients with the mild-moderate disease (0·55, p=0·02; 0·89, p<0·0001), simple snorers (0·34, p=0·0006; 0·58, p<0·0001), patients treated with CPAP (0·35, p=0·0008; 0·64, p<0·0001), and healthy participants (0·3, p=0·0012; 0·45, p<0·0001)
Li et al., 2014 [30]	Meta-analysis	2,851	Patients with OSA had a 31% greater risk of AF recurrence after successful catheter ablation compared to non-OSA patients (relative risk: 1.31, p=0.00). The risk of AF recurrence increased by 57% in patients who did not undergo CPAP therapy (OSA-CPAP patients) (relative risk: 1.57, p=0.00); however, patients who underwent CPAP therapy (OSA + CPAP) had a risk of AF recurrence similar to that of the non-OSA patients (relative risk: 1.25, p=0.59)
Qureshi et al., 2015 [29]	Meta-analysis	4,516	Eight studies published between 2003-2013 were included in the meta-analysis; 4,516 participants with AF underwent cardiac ablation, and out of these, 1,247 were diagnosed with OSA, and 698 were treated with CPAP. The study showed that the use of CPAP was associated with a 44% decreased risk of recurrence of AF (pooled relative risk: 0.56; 95% CI: 0.47-0.68; p<0.001)
Holmqvist et al., 2015 [34]	Observational	10,132	No major differences in hospitalization or cardiovascular outcomes were observed between patients with OSA with or without CPAP treatment; however, AF progression was less common in patients treated with CPAP
Shukla et al., 2015 [28]	Meta-analysis	1,087	Seven observational studies were incorporated. It was noted that users of CPAP had a significant reduction in AF recurrence rate compared with nonusers [n = 186 of 557 (33.3%) vs. 308 of 530 (58.1%); relative risk: 0.58; 95% CI: 0.51-0.67; p<0.001]. Additionally, In patients who underwent PVI, users of CPAP were found to have a lower risk of AF recurrence in comparison with nonusers [n = 173 of 518 (33.3%) vs. 273 of 474 (57.6%); relative risk: 0.58; 95% CI: 0.50-0.67; p<0.001]
Wu et al., 2016 [47]	Observational	72	CPAP therapy resulted in a significant decrease in the average number of episodes of heart block, from 148.58 ±379.44 to 16.07 ±58.52

## Conclusions

Many studies have demonstrated a correlation between OSA and decreased recurrence of AF with CPAP therapy, providing strong evidence that patients with OSA may be at a higher risk of AF and that treatment of OSA may help manage AF in this population. However, there is insufficient data available regarding the association between other atrial arrhythmias and OSA. Most published studies demonstrating a correlation between OSA, atrial arrhythmias, and CPAP therapy are primarily observational studies performed on small study populations. Large randomized clinical trials are needed to further endorse and confirm the relationship between OSA and atrial arrhythmias and the effects of CPAP use. Furthermore, additional randomized clinical trials are required to evaluate the benefit of other treatment modalities, such as autonomic nervous system modulation and risk-factor modifications, in patients with OSA and arrhythmias.
